# Incidence rate of mortality and its predictors among tuberculosis and human immunodeficiency virus coinfected patients on antiretroviral therapy in Ethiopia: systematic review and meta-analysis

**DOI:** 10.3389/fmed.2024.1333525

**Published:** 2024-04-19

**Authors:** Nebiyu Mekonnen Derseh, Muluken Chanie Agimas, Fantu Mamo Aragaw, Tilahun Yemanu Birhan, Solomon Gedlu Nigatu, Meron Asmamaw Alemayehu, Tigabu Kidie Tesfie, Tirualem Zeleke Yehuala, Tilahun Nega Godana, Mehari Woldemariam Merid

**Affiliations:** ^1^Department of Epidemiology and Biostatistics, Institute of Public Health, College of Medicine and Health Sciences, University of Gondar, Gondar, Ethiopia; ^2^Department of Health Informatics, Institute of Public Health, College of Medicine and Health Sciences, University of Gondar, Gondar, Ethiopia; ^3^Department of Internal Medicine, School of Medicine, University of Gondar Comprehensive Specialized Hospital, College of Medicine and Health Sciences, University of Gondar, Gondar, Ethiopia

**Keywords:** incidence rate, mortality, tuberculosis and human immunodeficiency virus coinfection, antiretroviral therapy, systematic review and meta-analysis, Ethiopia

## Abstract

**Background:**

Tuberculosis (TB) is the leading cause of death among HIV-infected adults and children globally. Therefore, this study was aimed at determining the pooled mortality rate and its predictors among TB/HIV-coinfected patients in Ethiopia.

**Methods:**

Extensive database searching was done via PubMed, EMBASE, SCOPUS, ScienceDirect, Google Scholar, and Google from the time of idea conception on March 1, 2023, to the last search via Google on March 31, 2023. A meta-analysis was performed using the random-effects model to determine the pooled mortality rate and its predictors among TB/HIV-coinfected patients. Heterogeneity was handled using subgroup analysis, meta-regression, and sensitivity analysis.

**Results:**

Out of 2,100 records, 18 articles were included, with 26,291 total patients. The pooled incidence rate of mortality among TB/HIV patients was 12.49 (95% CI: 9.24–15.74) per 100 person-years observation (PYO); I^2^ = 96.9%. The mortality rate among children and adults was 5.10 per 100 PYO (95% CI: 2.15–8.01; I^2^ = 84.6%) and 15.78 per 100 PYO (95% CI: 10.84–20.73; I^2^ = 97.7%), respectively. Age ≥ 45 (pooled hazard ratios (PHR) 2.58, 95% CI: 2.00– 3.31), unemployed (PHR 2.17, 95% CI: 1.37–3.46), not HIV-disclosed (PHR = 2.79, 95% CI: 1.65–4.70), bedridden (PHR 5.89, 95% CI: 3.43–10.12), OI (PHR 3.5, 95% CI: 2.16–5.66), WHO stage IV (PHR 3.16, 95% CI: 2.18–4.58), BMI < 18.5 (PHR 4.11, 95% CI: 2.28–7.40), anemia (PHR 4.43, 95% CI: 2.73–7.18), EPTB 5.78, 95% CI: 2.61–12.78 significantly affected the mortality. The effect of TB on mortality was 1.95 times higher (PHR 1.95, 95% CI: 1.19–3.20; I^2^ = 0) than in TB-free individuals.

**Conclusions:**

The mortality rate among TB/HIV-coinfected patients in Ethiopia was higher compared with many African countries. Many clinical factors were identified as significant risk factors for mortality. Therefore, TB/HIV program managers and clinicians need to design an intervention early.

## Background

Human immunodeficiency virus (HIV) infection weakens the immune system and increases the risk of tuberculosis (TB) in people living with HIV (PLHIV) (1). Tuberculosis is the leading opportunistic infection among PLHIV worldwide (2). Evidence shows that the probability of developing active TB among PLHIV is 18 times higher than among HIV-negative people (3).^1^ The WHO African region is the area with the highest HIV prevalence and a new episode of TB among PLHIV in the world (4). Ethiopia is one of the 30 countries with the highest TB burden in the world, and HIV infection is one of the driving factors behind this problem. In Ethiopia, the pooled prevalence of TB/HIV coinfection was 25.59% (5), and its incidence rate was 4.3 and 5 per 100 PYO among adults and children, respectively (6).

Tuberculosis was the leading cause of death from a single infectious agent, ranking above HIV/AIDS in the world (4). Tuberculosis-HIV coinfection is a serious global public health problem (7), and it is the main cause of death among PLHIV, accounting for around one-third of AIDS-related deaths in the world. According to the global TB report, there were an estimated 209, 000 (3), 214, 000 (8), 187, 000 (4) and 167, 000 (9) deaths of TB/HIV-coinfection in 2019, 2020, 2021, and 2022 respectively, which seems slightly decreasing but is a major cause of death among PLHIV. Out of the global TB/HIV deaths, 51%, 38%, and 11% were in men, women, and children, respectively, in 2022 (4). The global target for reducing TB/HIV deaths in 2020 was missed (62% vs 75%) (see text footnote ^3^).

The WHO African and Southeast Asia regions accounted for 82% of the total combined deaths of TB in HIV-negative and HIV-positive people in 2021 (4). For this reason, delay in ART initiation and the high prevalence of HIV might be two of the contributing factors (10). Studies in various African countries showed that the risk of death among TB/HIV-coinfected patients was higher than among TB-free HIV patients (11–15).

Tuberculosis and HIV-coinfected patients have an increased probability of death because of the bidirectional impact of TB and HIV (1, 13, 15–17). Tuberculosis facilitates HIV replication and leads to HIV viremia and severe immunosuppression; this might also be the main reason for the dissemination of TB in multiple organs, and HIV and ART also complicate ant-TB treatment. Evidence shows that there is an increased mortality rate in the early phase of anti-TB treatment (18) that could be due to TB associated immune reconstitution inflammatory syndrome (TB-IRIS) (19) and adverse drug reactions. The main reasons behind TB/HIV-coinfected deaths were late diagnosis, the impact of HIV on the clinical presentation, the diagnosis, drug-drug interactions, adverse drug reactions, IRIS, drug interruption, and LTFU. According to previous studies, age, being male, low CD4 cells, presence of OIs, anemia, occurrence of hepatotoxicity, being bedridden, not on CPT, HIV not disclosed, having EPTB, low BMI, and poor drug adherence (20–23) were independent predictors of death among TB/HIV coinfected patients.

In Ethiopia, despite the improvement of ART coverage, implementation of TPT, and TB/HIV collaborative activities, the TB/HIV-coinfection rate and its death rate are not reducing. Different scholars reported the mortality rate of TB/HIV in Ethiopia (22–27). However, there was no systematic review and meta-analysis (SRMA) study on the mortality rate of TB/HIV-coinfected patients in Ethiopia. A systematic review and meta-analysis study gives quality evidence of the pooled death rate among adults and children as well as the regional mortality rate and its predictors. It also shows the best evidence with fewer biases about the mortality rate of TB/HIV-coinfected patients and its predictors. This evidence could help policymakers, HIV/TB program managers, and clinicians design interventions, as well as for further monitoring and evaluation purposes. Therefore, this study aimed at determining the mortality rate and identifying its predictors among TB/HIV-coinfected patients in Ethiopia.

## Methods

### Searching strategy

We performed an SRMA based on the Preferred Reporting Items for Systematic Review and Meta-Analysis (PRISMA) 2020 guidelines (28). Electronic databases used for searching were PubMed, Scopus, Embase, and ScienceDirect, and websites like Google Scholar from the idea conception of March 1, 2023, to the last search via Google on March 31, 2023. We used synonyms under the MeSH term for the mortality rate. The entry terms with the combination we used for each searching database are shown in the Supplementary Table 1.

### Article selection and eligibility criteria

Either retrospective or prospective cohort studies that were reported, like time to death and/or hazard ratios among TB/HIV coinfected patients of all ages in Ethiopia and published between 2010 and 2023, were eligible for this study. All studies that fulfilled either incidence density or predictors with hazard ratios (HR) were included. In contrast, studies that were conducted among TB-free PLHIV and DR-TB/HIV patients in the absence of time and reported OR rather than HR were excluded from this study. Eligibility criteria were decided by all authors independently first, and then the agreement was taken with discussion when a conflict happened.

### Outcomes of the study

The primary outcome of interest was the pooled incidence rate of mortality among TB/HIV coinfected patients, which was measured by death (the event of interest) divided by the total person-years of observation. The second outcome of interest was pooled HRs for risk factors of mortality.

### Data extraction

All articles identified in each database were exported to the EndNote software, and then all duplicates were removed. Each article was screened independently by all authors with a title, abstract, and full text to identify eligible articles. Conflicts and differences were resolved by discussing them together. Data for each included study were extracted independently by NMD and MWM as the name of the first author, date of publication, study setting, target population, study region, study area, study design, sample size, median age, outcome of interest (death), PYO, incidence rate per 100 PYO, and predictors (HR and their 95% CI) by using standardized data extraction formats. The incidence rate (IR) per 100 PYO, log IR, SelogIR, and logHR with their standard errors were generated in MS Excel. Finally, all authors validated the data extraction formats.

### Data analysis and synthesis

A systematic review was conducted to review and summarize the primary studies by three authors (NMD, MWM, and MVA). The extracted data in MS Excel were imported to STATA 17 for meta-analysis. A meta-analysis was conducted to determine the overall pooled mortality rate and its predictors among TB/HIV-coinfected patients in Ethiopia using STATA 17 with the “metan” command. Heterogeneity was assessed both visually (the ‘eyeball' test) and statistically using the Cochran Q test or the I-squared statistic. The magnitude of statistical heterogeneity between studies was assessed using I^2^ statistics, and values of 25, 50, and 75% were considered to represent low, medium, and high, respectively. Because of the heterogeneity effect between studies, we performed a random-effects inverse-variance model with a DerSimonian-Laird estimate of tau^2^ for each of the included articles when more than one outcome of interest was available. The pooled mortality rate and 95% CI with I^2^ were displayed as summary effect estimates in a table and a forest plot in a figure for each article in random effect analysis. Sub-group analysis was conducted by using the study population, study region, and sample size category. We also performed meta-regression to handle heterogeneity. Moreover, we performed a sensitivity analysis to evaluate the key studies that exert a major impact on between-study heterogeneity. Publication bias was assessed by the funnel plot and Egger's regression tests.

## Results

### Searching results and included studies

**Figure 1:**
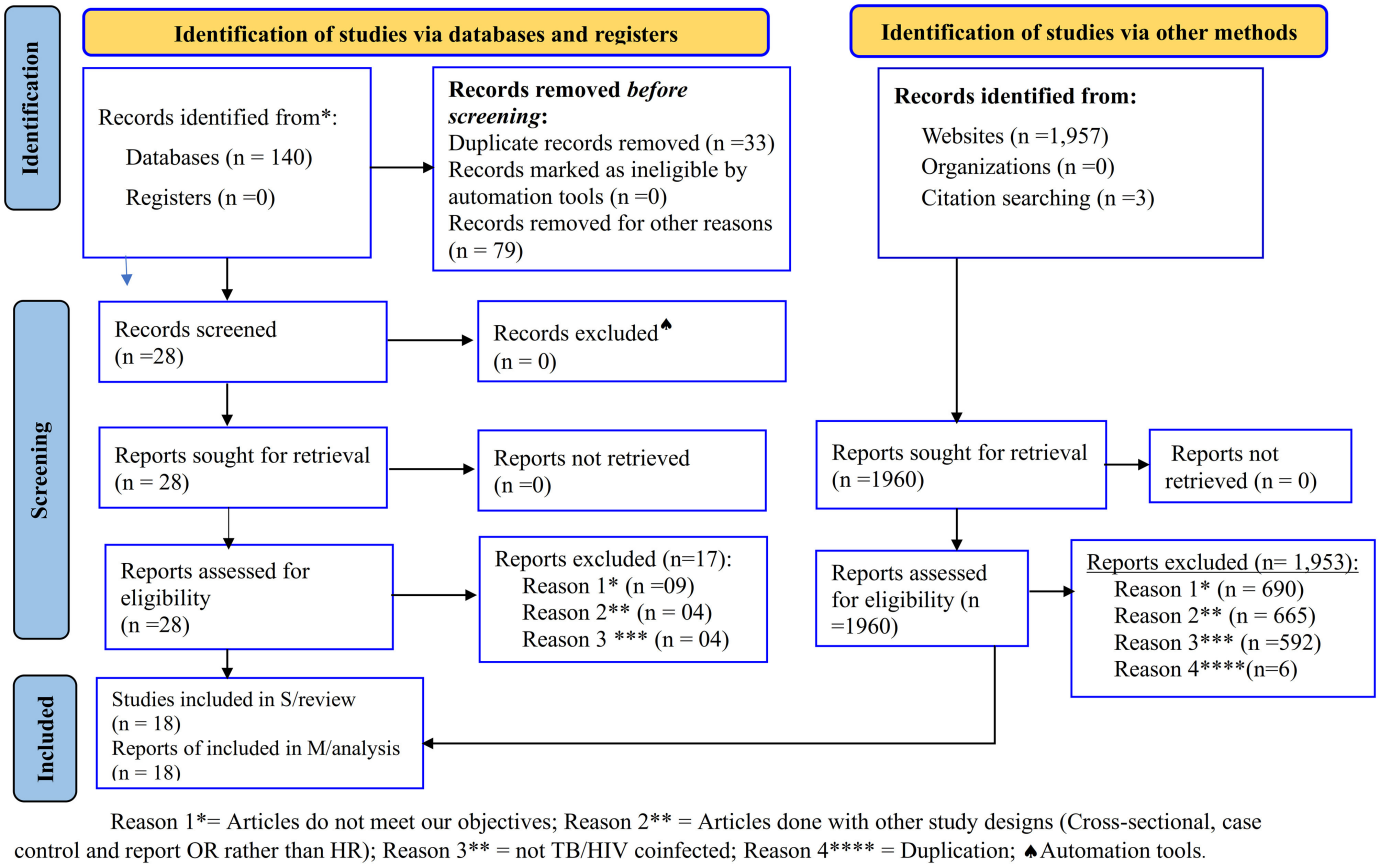
PRISMA flow chart for the studies screened, reviewed, and included (28).

A total of 2,100 articles were identified from all electronic databases and website searches. First, in all electronic databases, a total of 140 studies were exported to EndNote. Of these, 33 and 79 records were removed because of duplication and studies that did not meet our objectives, respectively, before conducting screening. After screening 28 studies by title, abstract, and/or full text, 17 studies were removed because they did not meet inclusion criteria. From the electronic database searches, a total of 11 articles (12, 22–26, 29–33) were selected for SRMA. Second, in the website search, a total of 1,960 records were identified. Out of these, 1,947 studies were removed because they did not meet the study objectives and study design, and the study populations were not TB/HIV-coinfected. Then, in the website search, out of 13 studies (17, 22–24, 26, 27, 32–38), six of them were removed because of duplication with previous electronic database searches, and as a result, seven studies were selected. Finally, a total of 18 articles (12, 17, 22–27, 29–38) with a total sample size of 26,291 TB/HIV-coinfected patients and non-TB cases as unexposed groups (two studies) were included in the SRMA study from both electronic databases and websites (Figure 1).

### Quality assessment

Three reviewers (NMD, MWM, and MCA) performed the quality assessment. A quality study was assessed using Newcastle-Ottawa quality assessment for cohort studies^2^^,^^3^ to assess the methodological quality and risk of bias. Each study was evaluated based on a maximum of one star for each numbered item within the selection and outcome categories, and a maximum of two stars was considered for comparability for the final decision of good, fair, and poor quality. Therefore, all selected studies had a score of good quality and were included in the analysis (Supplementary Table 2).

### Characteristics of included studies

**Figure 2:**
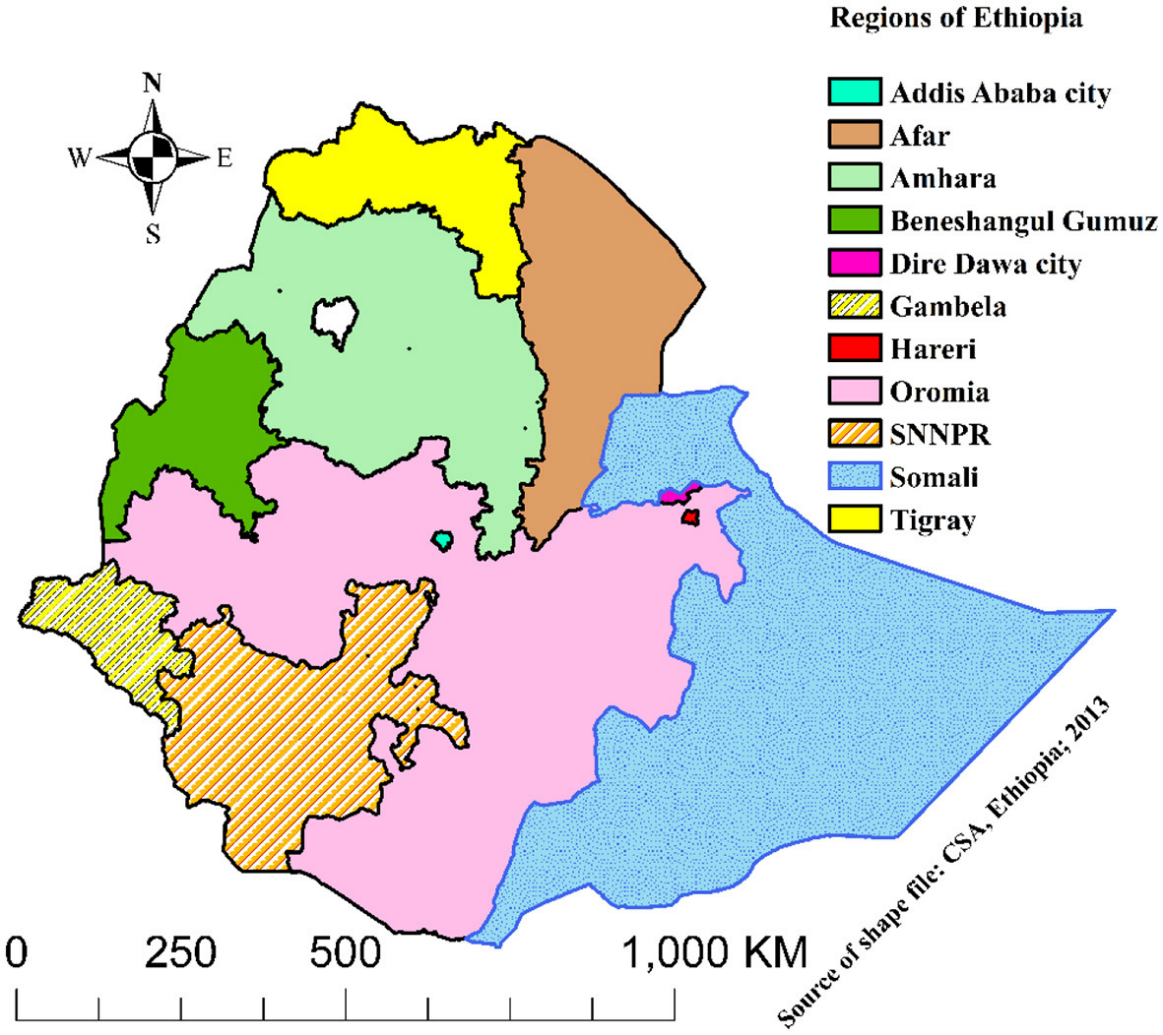
Map of Ethiopia with its regional classifications.

The characteristics of the included studies are summarized in Table 1. All of the studies were retrospective cohort studies that were published between 2010 and 2023 in English. Studies were conducted in five regions (Tigray, Amhara, Oromia, SNNPR, and Harari) of Ethiopia and two city administrations (Addis Ababa and Dire-Dawa) as shown in the map below (Figure 2). All of the studies were done in public health facilities, including health centers, primary hospitals, and comprehensive specialized hospitals, as a multi-center study. All of the included study populations were TB/HIV-coinfected except for two studies, which were TB/HIV-coinfected cases as exposed and non-TB cases as an unexposed group. The maximum and minimum sample sizes were 16,061 (29) and 227 (26), respectively. The highest and lowest prevalence of death were 35.80% in Debre Tabor, Amhara (33) and 1.81% in Addis Ababa (29), respectively. The highest and lowest incidence rates were 61.10 (31) and 1.22 (25) per 100 PYO in SNNPR and Addis Ababa, respectively. Half of the studies reported the median time to death (Table 1).

**Table 1:** Characteristics of included studies (*n* = 16).

**References**	**Study region**	**Study setting**	**Length FUP period Mo**	**Study population**	**Sample size**	**Proportion**	**Death**	**Total PYO**	**IR/100 PYO**
Gemechu et al. (23)	SNNPR	Hawassa, Walayta	131	Children	284	12.32	35	1,257.17	2.78
Atalell et al. (27)	Amhara	UoG C/Specialized Hospital	145	Children	271	14.02	38	1,167.67	3.25
Chanie et al. (26)	Amhara	Northwest Ethiopia Hospitals	82.36	Children	227	17.18	39	1,063.2	3.67
Nigusie J. et al. (38)	Tigray	Mekelle, Alamata, and Maych	120	Children	253	15.02	38	210.98	18.01
Dawit et al. (36)	SNNPR	Southern Ethiopia	119.99	children	274	17.15	47	1,581.3	2.97
Abrha et al. (32)	Oromia	Jima U/C/specialized Hospital	23.99	Adults	272	20.22	55	253.37	21.71
Birhan et al. (33)	Amhara	Debre Tabor PHF	71	Adults	243	35.80	87	521.41	16.69
Gezae et al. (22)	Tigray	Ayder C/Sp/H/& Mekelle HC	95	Adults	305	22.95	70	980.77	7.14
Habtegiorgis et al. (24)	Dire-Dawa	Dire-Dawa PHF	59	Adults	471	16.77	79	945.13	8.36
Kassa et al. (29)	Addis A.	Zewditu Memorial Hospital	48	Adults	270	21.11	291	10,392.85	2.80
Lelisho et al. (34)	SNNPR	Mizan-Tepi C/Sp/Hospital	60	Adults	363	21.76	79	754.82	10.47
Lelisho et al. (35)	SNNPR	Mettu Karl Referral Hospital	95.95	Adults	402	20.90	84	568.75	14.77
Refera (30)	Oromia	Ambo C/specialized Hospital	65.92	Adults	501	15.77	79	823.54	9.59
Seyoum et al. (25)	Addis A.	AA public facilities	84	Adults	1,123	4.45	50	4,114.09	1.22
Sime et al. (12)	Harar & Dire-Dawa	H/Fana, Jugal, D/Chora Hospitals	53.95	Adults	566	10.60	60	530.6	11.31
Teklu et al. (17)	Addis A.	seven hospitals	96	Adults	3,889	10.05	391	15,038.46	2.60
Wondimu et al. (31)	SNNPR	Mizan Tepi C/Sp/hospital	119.95	Adults	364	22.80	83	135.94	61.06
Sileshi et al. (37)	Amhara	Bahir Dar Public health facility	33.99	Adults	422	22.04	93	186.93	49.75

### National pooled mortality rate among TB/HIV coinfected patients

**Figure 3:**
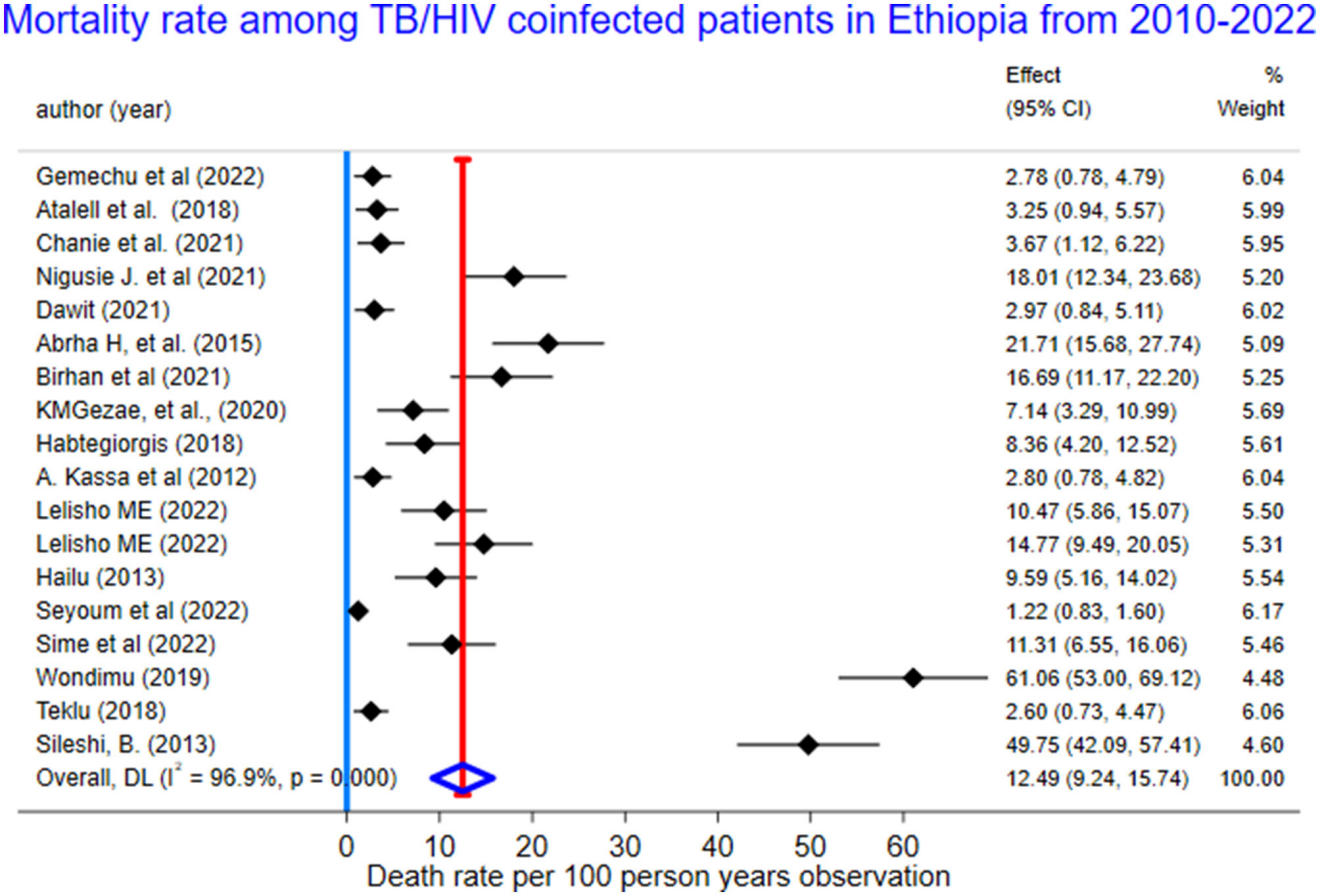
The forest plots for mortality rate among TB/HIV-coinfected patients in Ethiopia, 2010–2023.

We used a random-effects inverse-variance model with a DerSimonian-Laird estimate of tau^2^ to determine the overall pooled mortality rate among TB/HIV-coinfected patients in Ethiopia from 2010 to 2023. The pooled cumulative incidence of mortality was 17% (95% CI: 13–20%; I^2^ = 98.6%), while the pooled incidence rate of mortality was 12.49 per 100 PYO (95% CI: 9.24–15.74). The heterogeneity was significantly high (I^2^ = 96.9%, 95% CI: 72.6–98.9) based on Cochran's Q value of 552.18, and the *P*-value was < 0.001. This showed that the proportion of total variation in the effect estimate was due to between-study heterogeneity (Table 2, Figure 3).

**Table 2:** Pooled mortality rate among TB/HIV-co-infected patients in Ethiopia from 2010 to 2023.

**References**	**Effect**	**95% CI**	**Weight%**	**Cochran's Q statistics**	**I^2^ %**	***P*-value**
Gemechu et al. (23)	2.78	0.78–4.79	6.04			
Atalell et al. (27)	3.25	0.94–5.57	5.99			
Chanie et al. (26)	3.67	1.12–6.22	5.95			
Nigusie J. et al. [2021]	18.01	12.35–23.68	5.20			
Dawit et al. (36)	2.97	0.84–5.11	6.02			
Abrha et al. (32)	21.71	15.68–27.74	5.09			
Birhan et al. (33)	16.69	11.17–22.20	5.25			
Gezae et al. (22)	7.14	3.29–10.99	5.69			
Habtegiorgis et al. (24)	8.36	4.20–12.52	5.61			
Kassa et al. (29)	2.80	0.78–4.82	6.04			
Lelisho et al. (34)	10.47	5.86–15.07	5.50			
Lelisho et al. (35)	14.77	9.49–20.05	5.31			
Hailu [2013]	9.59	5.16–14.02	5.54			
Seyoum et al. (25)	1.22	0.83–1.60	6.17			
Sime et al. (12)	11.31	6.55–16.06	5.46			
Wondimu et al. (31)	61.06	53.00–69.12	4.48			
Teklu et al. (17)	2.60	0.73–4.47	6.06			
Sileshi et al. (37)	49.75	42.09–57.41	4.60			
Pooled incidence of mortality per 100 person years observation	12.49	9.24–15.74	100.00	552.18	96.9, 95% CI: 72.6–98.9	<0.001

### Handling heterogeneity

#### Subgroup analyses

We performed subgroup analysis using predetermined parameters to compare the pooled mortality rate (PMR) with the sub-group values. We conducted it based on the region, study population, and sample size category. Using sub-group analysis based on the region, the PMR in southern Ethiopia (SNNPR) was 17.64 per 100 PYO (95% CI: 6.78–28.50; Q = 211.05, I^2^ = 98.1%, *p* < 0.001), while the PMR in eastern Ethiopia (Harar and Dire-Dawa) was 11.31 per 100 PYO (95%: 6.55–16.06; Q and I^2^ = 0) (Supplementary Table 4, Supplementary Figure 1).

**Figure 4:**
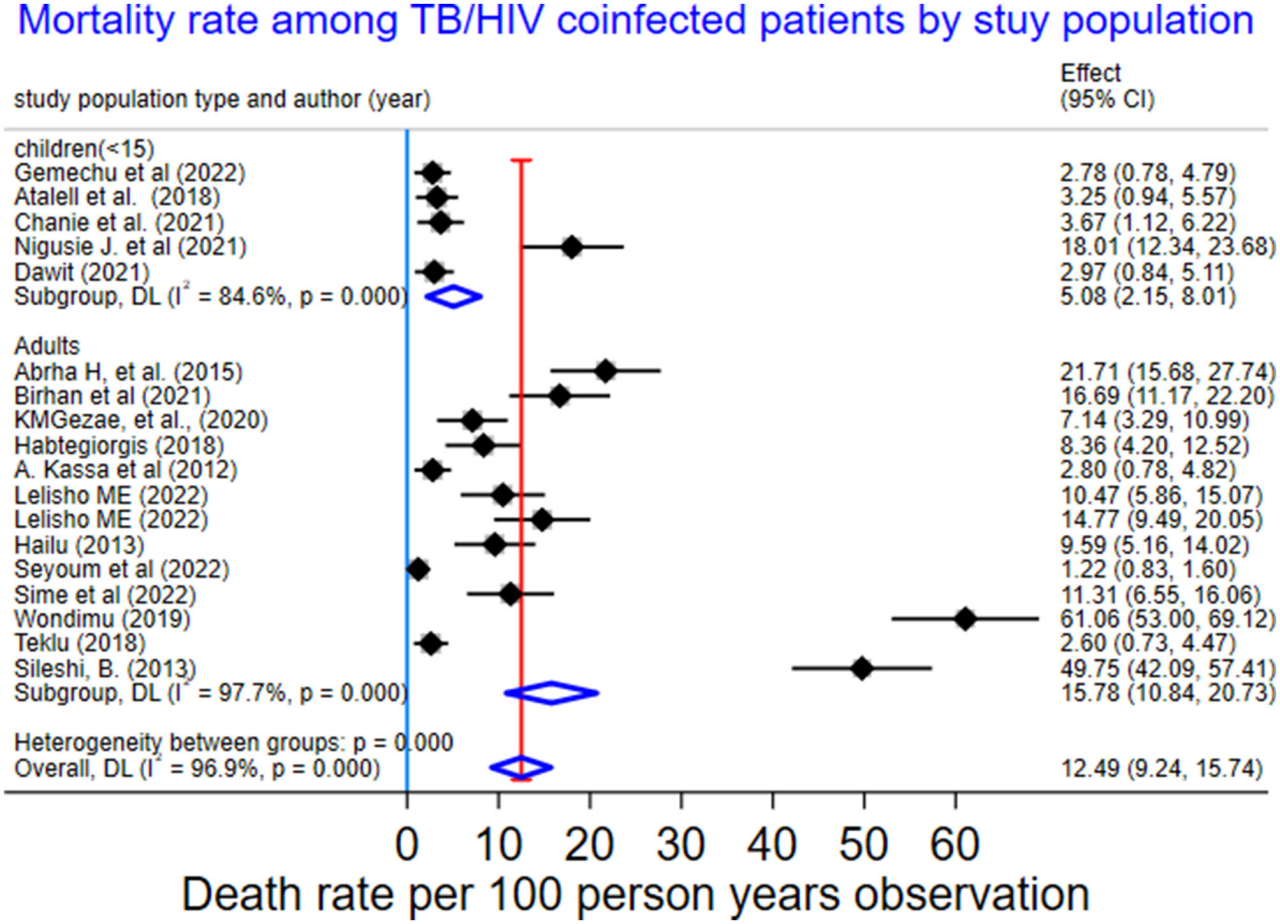
Subgroup-analysis of mortality rate by study population among TB/HIV-coinfected patients in Ethiopia, 2010–2023.

We also performed a sub-group analysis by using the study population. Accordingly, the PMR among under-fifteen-year-old children was 5.08 per 100 PYO (95% CI: 2.15–8.01; Q = 25.89, I^2^= 84.6%, and *p* < 0.001). In contrast, the PMR among adults aged 15 years or older was 15.78 per 100 PYO (95% CI: 10.84–20.73; Q = 518.36, I^2^= 97.7%, and *P* < 0.001). Despite subgroup analysis, heterogeneity was a little bit higher (Table 3, Figure 4).

**Table 3:** Subgroup-analysis of mortality rate among TB/HIV-coinfected patients by study population in Ethiopia, 2010 to 2023.

**Study population**	**References**	**Effect**	**95% CI**	**Wt%**	**Q-statist**	**I^2^ %**	***p*-value**
Under 15 years Children	Gemechu et al. (23)	2.78	0.78–4.79	6.04			
	Atalell et al. (27)	3.25	0.94–5.57	5.99			
	Chanie et al. (26)	3.67	1.12–6.22	5.95			
	Nigusie J. et al. [2021]	18.01	12.35–3.68	5.20			
	Dawit et al. (36)	2.97	0.84–5.11	6.02			
	Subgroup, DL	5.08	2.15–8.01	29.21	25.89	84.6	<0.001
Adults ≥15 years	Abrha et al. (32)	21.71	15.68–7.74	5.09			
	Birhan et al. (33)	16.69	11.17–2.20	5.25			
	Gezae et al. (22)	7.14	3.29–10.99	5.69			
	Habtegiorgis et al. (24)	8.36	4.20–12.52	5.61			
	Kassa et al. (29)	2.80	0.78–4.82	6.04			
	Lelisho et al. (34)	10.47	5.86–15.07	5.50			
	Lelisho et al. (35)	14.77	9.49–20.05	5.31			
	Hailu [2013]	9.59	5.16–14.02	5.54			
	Seyoum et al. (25)	1.22	0.83–1.60	6.17			
	Sime et al. (12)	11.31	6.55–16.06	5.46			
	Wondimu et al. (31)	61.06	53.00–9.12	4.48			
	Teklu et al. (17)	2.60	0.73–4.47	6.06			
	Sileshi et al. (37)	49.75	42.09–7.41	4.60			
	Subgroup, DL	15.78	10.84–20.73	70.79	518.36	97.7	<0.001
Pooled death rate per 100-PYO		12.49	9.24–15.74	100.00	552.18	96.9	<0.001

Subgroup analysis was also conducted based on the sample size category in the primary studies. The PMR for the sample size lower than 500 was 15.11 per 100 PYO (95% CI: 10.08–20.13; Q statis = 413.71, I^2^ = 96.6%, and *P* < 0.001). In contrast, the PMR for sample sizes greater than 500 was 5.36 per 100-PYO (95% CI: 1.83–8.88, Q statistics = 32.29, I^2^ =90.7%, P-value < 0.001) (Supplementary Table 5, Supplementary Figure 2).

#### Meta-regression

We computed meta-regression for sex (male), functional status (ambulatory and bedridden), WHO clinical staging (stage III and IV), and CPT (no); the I^2^ ranged from 11 to 84% (Table 4).

**Table 4:** Meta-regression for selected variables among TB/HIV-coinfected patients in Ethiopia from 2010 to 2023.

**Sr. No**.	**Variables**	**Categories**	**HR**	***P*-value**	**[95% CI]**	**I^2^ %**	**tau^2^**	**Number of studies**
1.	Sex	Female	Ref					
		Male	2.99	0.035	2.65–3.37	65.77	0	13
2.	Functional status	Working	Ref					
		Ambulatory	3.13	<0.001	2.93–3.46	35.77	0	11
		Bedridden	6.70	0.248	3.10–14.48	83.36	0.82	11
3.	WHO clinical stage	Stage I & II	Ref					
		Stage III	3.05	0.001	1.82–5.10	63.84	0.24	10
		Stage IV	4.17	<0.001	2.20–7.95	83.84	0.75	15
4.	CPT	NO	6.08	0.01	3.98– 9.29	11.78	0.07	11
		Yes	Ref					

#### Sensitivity analysis

We conducted a leave-one-out sensitivity analysis to identify and minimize the source of heterogeneity. When we dropped one study that was an outlier, the PMR was increased to 13.62 per 100-PYO (95%: 9.53–17.72; I^2^ = 96.3%, Q = 433.99, and *P* < 0.001) (Supplementary Table 6, Supplementary Figure 3).

### Publication bias

We performed a publication bias both graphically and statistically. The graph of the funnel plot appeared asymmetrically. Likewise, Egger's test for small-study effects showed that there was evidence of publication bias (coefficient = 5.60, *p* ≤ 0.001) (Supplementary Table 5).

### Pooled predictors for TB/HIV coinfected death

We conducted a meta-analysis for potential predictors using the random-effects inverse-variance model to identify the pooled HR and its 95% CI. TB/HIV-coinfected patients aged 45 years or older had a 2.58 times higher risk of death compared with those aged 15–24 years (pooled hazard ratio (PHR): 2.58, 95% CI: 2.00–3.31; I^2^ = 85.5%). Similarly, the hazard of death among unemployed coinfected patients was 2.17 times higher than that of government employees (PHR = 2.17, 95% CI: 1.37–3.46; I^2^ =49.7%). The hazard of mortality among HIV status non-disclosed was 2.79 times higher compared with their counterparts (PHR = 2.79, 95% CI: 1.65–4.70; I^2^ = 49.7%). The survival status was 5.89 times higher among bedridden compared with working functional status (PHR = 5.89, 95% CI: 3.43–10.12; I^2^ =84.9%). The risk of death among coinfected patients with WHO clinical stages III and IV was 2.85 and 3.16 (PHR = 2.85, 95% CI: 1.97–4.13, I^2^ = 63.8 and PHR = 3.16, 95% CI: 2.18–4.58, I^2^ = 83.8), respectively. Similarly, the hazard of death among anemic coinfected patients was 4.43 times higher than their counterparts (PHR = 4.43, 95% CI: 2.73–7.18; I^2^ = 44.8%). Coinfected patients with a BMI < 18.5 were 4.11 times (PHR = 4.11, 95% CI: 2.28–7.40; I^2^ = 96.8%) higher than those with a BMI ≥ 18.5. The hazard of mortality among TB/HIV-coinfected patients with a CD4 count <50 cells/mm^3^ was 1.54 times higher compared with a CD4 count ≥ 200 cells/mm^3^ (PHR = 1.54, 95% CI: 1.11–2.14; I^2^ = 0%). In this study, the effect of TB on mortality was almost twice higher compared with TB-free individuals (PHR = 1.96; 95% CI: 1.19–3.20; I^2^ = 0%). Similarly, the hazard of death among coinfected patients with EPTB was 5.78 times (PHR = 5.78, 95% CI: 2.61–12.78; I^2^ = 95.3%). Similarly, individuals who did not take CPT were 1.65 times more likely to die compared with their counterparts (PHR = 1.65, 95% CI: 1.22–2.23; I^2^ = 0%) (Table 5).

**Table 5:** Meta-analysis for pooled predictors of mortality rate among TB/HIV-coinfected patients in Ethiopia, 2010–2023.

**Sr. No**.	**Variables**	**Categories**	**Pooled HR**	**95% CI**	**I^2^ (%)**	**Cochran's Q stat**	***P*-Value for Q**
1.	Residence	Rural	1.38	0.40–4.75	87.0	38.46	<0.001
		Urban	Ref				
2.	Sex	Male	1.06	0.94–1.2	16.4	14.36	0.278
		Female	Ref				
3.	Marital status	Single	Ref				
		Married	1.10	0.89–1.36	18.9	2.47	0.29
		Divorced	1.74	0.84–3.59	96.9	31.82	<0.001
4.	Age	<1 year	0.96	0.19–4.79	0	0.22	0.894
		1-5 year	0.91	0.78–1.08	0	0.11	0.947
		6-10 years	1.20	0.56–2.61	81	10.55	0.005
		10–15	Ref	0.778			
		15–24	Ref				
		25–34	1.23	0.83–1.81	70.9	20.63	0.002
		35–44	0.87	0.65– 1.16	88.7	44.16	0.051
		≥45	2.58^*^	2.00– 3.31	84.5	32.32	<0.001
5.	Educational status	No-education	Ref				
		Primary	0.88	0.63–1.24	61.7	15.68	0.016
		Secondary	1.01	0.78–1.31	17.7	6.08	0.30
		Tertiary	1.01	0.78–1.31	17.7	6.08	0.30
6.	Occupation	Gov't Employee	Ref				
		Unemployed	2.17^*^	1.37–3.46	37.6	3.21	0.21
		NGO Employee	0.68	0.32– 1.47	0	0.49	0.49
		Farmer	0.69	0.36–1.33	0	0	0.97
		CSW	1.12	0.29–4.29	0	0.07	0.79
7.	Disclosure	No	2.79 ^*^	1.65–4.70	49.7	5.96	0.114
		Yes	Ref				
8.	Functional status	Working	Ref				
		Ambulatory	1.14	1.09–1.19	0	7.49	0.68
		Bedridden	5.89^*^	3.43–10.12	84.9	66.09	<0.001
9.	OI	NO	Ref				
		Yes	3.5^*^	2.16–5.66	99.9	12,771.44	<0.001
10.	WHO clinical staging	Stage I & II	Ref				
		Stage III	2.85	1.97–4.13	63.8	24.89	0.003
		Stage IV	3.16^*^	2.18–4.58	83.8	86.65	<0.001
11.	Undernutrition	BMI≥18.5	Ref				
		BMI < 18.5	4.11^*^	2.28–7.40	96.8	92.98	<0.001
12.	CD4 cells count	<50 cells/mm^3^	1.54^*^	1.11–2.14	0	1.48	0.915
		50–200c/mm^3^	1.63 ^*^	1.20 −2.22	0	1.28	0.733
		≥200 cells/mm^3^	Ref				
13.	Anemia	Hg >10 mg/dl	Ref				
		Hg < 10 mg/dl	4.43^*^	2.73–7.18	44.8	12.67	0.081
14.	Adherence	Good	Ref				
		Poor	1.11^*^	1.02–1.21	0	2.37	0.88
15.	Effect of TB	TB/HIV	1.95^*^	1.19–3.20	0	0.33	0.856
16.	Site of TB	PTB	Ref				
		EPTB	5.78^*^	2.61– 12.78	95.3	148.15	<0.001
17.	CPT	NO	1.65^*^	1.22–2.23	0	3.07	0.98
		Yes	Ref				

## Discussion

Despite the presence of effective HAART and TPT, the impact of TB on the mortality of PLHIV in Ethiopia was substantial (12, 25, 39, 40). Therefore, this study aimed to determine the mortality rate and its predictors among TB/HIV-coinfected patients in Ethiopia using SRMA.

The pooled mortality rate of 12.49 (95% CI: 9.24–15.74) per 100 PYO among TB/HIV-coinfected patients in Ethiopia was consistent with 10.1 per 100 PYO in South Africa (41) and 9.44 per 100 PYO in China (42). On the other hand, this study was higher than studies done in Cameroon (32.2 per 100 PMO) (18), Mozambique (6.8 per 100 PY) (43), Uganda (15.42 per 1,000 PY) (44), and England (2.13 per 100 PY) (45). The variation could be because of differences in the follow-up time of the included study studies, variation in study population, the number of studies, socio-demographic, and clinical differences.

In this study, subgroup analysis showed that the death rate in children under 15 with TB/HIV was 5.1 (95% CI: 2.15–8.01) per 100 PYO, which was higher than a study in Nigeria's 1.4 per 100 PYO (20). The incidence rate of mortality among adults was 15.78 (95% CI: 10.84–20.73) per 100 PYOs, which was higher than that of under-15-year-old children. This could be attributed to differences in behavioral and clinical factors. Moreover, there could be a higher incidence rate of LTFU among adults (46) than children (47). In this study, the overall pooled incidence of mortality among TB/HIV patients was 17%, or 170 deaths per 1,000 people (95% CI: 130–200). This finding was in line with the WHO global TB reports for 2022 (187 deaths per 1,000 population) (4) and 2023 (167 deaths per 1,000 people) (9). In contrast to this, this study was lower than the WHO global TB report for 2020 (209 deaths per 1,000 population) (3) and 2021 (214 per 1,000 population) (8). The variation might be because of the impact of the COVID-19 pandemic, which aggravated mortality.

The pooled effects of different risk factors were significantly affecting the mortality of TB/HIV-coinfected patients in Ethiopia. The hazard of death among TB/HIV-coinfected patients aged 45 years or more was 58% higher compared with young adulthood. Evidence showed that as age increased by 10 years, the risk of mortality increased by 38% (48). This might be attributed to an increased likelihood of comorbidities as age increases. This finding was supported by a study conducted in Uganda (49), Botswana (21), and South Africa (14, 50).

Likewise, unemployed TB/HIV-coinfected patients were at a 2.17 times higher risk of dying than government employees. Existing evidence supports this finding (51, 52). This could be because unemployed patients would have a lack of money for transportation to the service facilities and for food, as well as an increased tendency to have substance abuse.

In the current study, TB-coinfected patients who did not disclose their HIV status were at a 79% higher risk of dying compared with their counterparts. This might be because HIV non-disclosure resulted in treatment interruption, poor adherence to medication, and an increased likelihood of LTFU from care and treatment. This finding was supported by a previous study (53).

Similarly, bedridden coinfected patients had 5.89 times a higher tendency for death compared with those with functional working status. This could be because of an increasing number of OIs and undernutrition in these patients. This was in line with existing evidence (53, 54).

This study found that the hazard of dying among TB/HIV-coinfected patients with OIs other than TB was 3.5 times greater than their counterparts. Studies in Malaysia (55) and Botswana (21). supported this finding. This might be attributed to synergistic effects with TB, which facilitated HIV replication, increased viremia, and immunosuppression. Moreover, there could be a high likelihood of ADRs, IRIS, and DDIs between ART, anti-TB, and OI medications, which would facilitate mortality (21, 56).

This meta-analysis study elucidated that TB-coinfected patients with advanced HIV disease had an increased risk of dying. Coinfected patients with WHO clinical stages III and IV were 2.85 and 3.16 times, respectively, at a higher risk of death than those with stage I. This was supported by previous studies (53, 57). Similarly, patients with advanced immunosuppression like CD4 cells 50–200 cells/mm^3^ and <50 cells/mm^3^ had a 1.63- and 1.54-times higher risk of mortality than those with adequate immunity. Various studies done in Uganda (44), Malawi (58), South Africa (15), Nigeria (20), Guinea-Bissau (59), Malaysia (55), Myanmar (54), and China (42, 60) supported this finding. Existing evidence showed that as CD4 increased by 50% among coinfected patients, the hazard of death decreased by 18% (48), and when CD4 cells were more than 350, the death rate decreased by 76% and more (50, 61).

In this study, the hazard of death among TB/HIV patients with anemia was 4.43 times higher than their counterparts. This is because of worsening immunosuppression and might be due to decreased oxygen saturation among patients with anemia. This was in line with existing findings (21, 57, 62, 63).

Our finding showed that undernourished coinfected patients were at a 4.11 times higher risk of death compared with those having adequate nutrition. This finding was consistent with previous studies (64, 65). This was because undernourished patients might have low immunity and be vulnerable to different OIs (66), a higher risk of having ADRs, and poor adherence, which leads to LTFU and death.

The pooled effect of three studies showed that the effect of TB on the mortality of HIV patients was 1.95 times higher than that of TB-free individuals. This could be mainly because of the bidirectional impact of TB/HIV, as described by many studies (11, 25, 50, 63, 67, 68). This finding was in line with a study in South Africa (HR of 2) (67) and a metal analysis study (HR of 1.8) (69). A study in the South Africa also showed that the hazard of death among TB/HIV-coinfection patients was 4.8 times higher than among non-coinfected patients (45). Evidence shows that TB results in immune cell activation (1), then aids in HIV replication, increasing viremia, and ultimately leading to immunosuppression and death.

This study showed that PLHIV patients with EPTB were at a 5.78 times higher risk of dying compared with PTB patients. This was consistent with studies in South Africa (14, 50), Cameroon (18), and China (42, 60). This might be because of the high risk of IRIS in ART-naïve patients, anemia, and the increased likelihood of having advanced HIV in these patients. Evidence also showed that HIV patients with EPTB have delayed diagnosis, which facilitates mortality (70).

The hazards of death among coinfected patients who did not take CPT were 1.65 times higher than their counterparts. A previous study supported this finding (62). Evidence also showed that taking CPT decreased HIV-associated mortality by 74% (63).

### Strengths and limitations

This systematic review and meta-analysis study includes both adults and children to determine the pooled mortality rate and its pooled predictors, which help HIV/TB program managers and other concerned bodies make decisions at the national level. Some scholars didn't report person-time observations, which was solved by contacting the corresponding authors. Most of the included primary studies had moderately small sample sizes, which were the main source of heterogeneity. Moreover, there were no primary studies conducted equally in all regions of Ethiopia.

## Conclusions and recommendations

The incidence rate of mortality among TB/HIV-coinfected people in Ethiopia was 1.22 per 100 PYO, the lowest in Addis Abebe, and 61.1 per 100 PYO, the highest in SNNPR. The pooled death rate of TB and HIV was higher than in many African and Asian countries.

Age of more than 45 years, being unemployed, HIV not disclosed, being bedridden, having anemia, WHO clinical stages III and IV, having EPTB, undernutrition, OIs, CD4 below 200 cells/mm^3^, and no CPT were significant risk factors for mortality. This study found that the effect of TB on mortality was substantial. Therefore, the government of Ethiopia and its stakeholders need to design interventions, especially focusing on older people, the unemployed, and those with advanced HIV disease. Clinicians could give their attention to facilitating HIV disclosure, early diagnosis, and management of OIs, undernutrition, anemia, and EPTB.

## Data availability statement

The original contributions presented in the study are included in the article/Supplementary material, further inquiries can be directed to the corresponding author.

## Author contributions

ND: Conceptualization, Data curation, Formal analysis, Investigation, Methodology, Software, Supervision, Validation, Visualization, Writing – original draft, Writing – review & editing, Project administration, Resources. MCA: Data curation, Formal analysis, Investigation, Methodology, Software, Supervision, Validation, Visualization, Resources, Project administration, Writing – original draft. FA: Data curation, Formal analysis, Investigation, Methodology, Validation, Visualization, Software, Resources, Supervision, Writing – original draft. TB: Data curation, Investigation, Supervision, Validation, Visualization, Formal analysis, Methodology, Project administration, Resources, Software, Writing – original draft. SN: Data curation, Investigation, Supervision, Validation, Methodology, Software, Formal analysis, Project administration, Resources, Writing – original draft. MAA: Data curation, Investigation, Supervision, Validation, Visualization, Formal analysis, Methodology, Software, Project administration, Resources, Writing – original draft. TT: Data curation, Investigation, Supervision, Validation, Visualization, Formal analysis, Methodology, Software, Project administration, Resources, Writing – original draft. TY: Data curation, Investigation, Methodology, Supervision, Validation, Visualization, Formal analysis, Software, Project administration, Resources, Writing – original draft. TG: Data curation, Investigation, Methodology, Supervision, Validation, Visualization, Formal analysis, Project administration, Resources, Software, Writing – original draft. MM: Data curation, Formal analysis, Investigation, Methodology, Software, Supervision, Validation, Visualization, Project administration, Resources, Writing – original draft.

## Footnotes

^1^ https://www.unaids.org/sites/default/files/media_asset/20220324_TB_FactSheet_en.pdf

^2^ https://www.ncbi.nlm.nih.gov/books/NBK115843/bin/appe-fm3.pdf

^3^ https://bmjopen.bmj.com/content/bmjopen/8/12/e023953/DC4/embed/inline-supplementary-material-4.pdf?download=true
