# Supplementary material for: Incidence rate of mortality and its predictors among tuberculosis and human immunodeficiency virus coinfected patients on antiretroviral therapy in Ethiopia: systematic review and meta-analysis
Source: Front Med (Lausanne). 2024 Apr 19;11:1333525. doi: 10.3389/fmed.2024.1333525 (PMC11066242; doi:10.3389/fmed.2024.1333525)
Supplement: Supplementary file 1 [file Data_Sheet_1.pdf]

Table Supl.1: Searching strategies for both electronic databases and websites to conduct systematic review and metanalysis of death rate among TB/HIV coinfected patients in Ethiopia, from 2010 to 2023.

MeSH Heading	Synonyms	Entry terms	Combination	# Of articles	Last date of searching	Electronic Data bases & webs
Mortality	Age Specific Death Rate; Age-Specific Death Rate; CFR Case Fatality Rate; Case Fatality Rate; Crude Death Rate; Crude Mortality Rate; Death Rate; Decline, Mortality; Determinants, Mortality; Differential Mortality; Excess Mortality; Mortality	Mortality rate OR Time to death OR Survival* OR Incidence of mortality OR Mortality AND Tuberculosis/HIV-coinfected OR TB-HIV co-infected OR TB/HIV co-infected OR HIV-TB coinfected OR HIV/TB coinfected OR co-infected with tuberculosis and human immunodeficiency virus OR TB and HIV-coinfected OR HIV-infected individuals with tuberculosis AND predictors OR determinant factors OR associated factors OR Factors affecting AND Ethiopia	(((((Mortality rate [MeSH Major Topic])) OR (Time to death OR Survival* OR "Incidence of mortality" OR Mortality [MeSH Terms])) AND (Tuberculosis/HIV-coinfected OR TB-HIV co-infected OR TB/HIV co-infected OR HIV-TB coinfected OR HIV/TB coinfected OR co-infected with tuberculosis and human immunodeficiency virus OR TB and HIV-coinfected OR HIV-	33	March 31, 2023	PubMed

	Decline; Mortality Determinants; Mortality Rate; Mortality, Differential; Mortality, Excess		infected individuals with tuberculosis [MeSH Terms])) AND (predictors OR determinant factors OR associated factors OR Factors affecting)) AND (Ethiopia)			
		Mortality rate OR Time to death OR Survival* OR "Incidence of mortality" AND “Tuberculosis-HIV-coinfected” OR TB-HIV co-infected OR HIV-TB coinfected OR "co-infected with tuberculosis and human immunodeficiency virus" OR “TB and HIV-coinfected” OR "HIV-infected individuals with tuberculosis" AND predictors AND Ethiopia	(((((mortality AND rate OR time) AND to AND death OR survival* OR 'incidence of mortality') AND 'tuberculosis-HIV-coinfected' OR 'tb HIV') AND 'co infected' OR 'HIV tb') AND coinfected OR 'co-infected with tuberculosis and human immunodeficiency virus' OR 'tb and HIV-coinfected' OR 'HIV-infected individuals with tuberculosis') AND predictors AND Ethiopia AND [2010-2023]/py	29	March 14, 2023	EMBASE

			By filtering publication date 2010-2023, studies conducted on humans and only English language			
		Mortality rate OR Time to death OR Survival* OR Incidence of mortality OR Mortality AND Tuberculosis/HIV-coinfected OR TB-HIV co-infected OR TB/HIV co-infected OR HIV-TB coinfected OR HIV/TB coinfected OR co-infected with tuberculosis and human immunodeficiency virus OR TB and HIV-coinfected OR HIV-infected individuals with tuberculosis AND predictors OR determinant factors OR associated factors OR Factors affecting AND Ethiopia		31	March 21, 2023	SCOPUS

		Mortality rate and its predictors among tuberculosis and human immunodeficiency virus coinfected patients on antiretroviral treatment in Ethiopia		47	March10, 2023	Science Direct
		Mortality rate OR Time to death OR Survival* OR "Incidence of mortality" AND “Tuberculosis/HIV-coinfected” OR TB-HIV co-infected OR TB/HIV co-infected OR HIV-TB coinfected OR HIV/TB coinfected OR "co-infected with tuberculosis and human immunodeficiency virus" OR “TB and HIV-coinfected” OR "HIV-infected individuals with tuberculosis" AND predictors OR “determinant factors” OR “associated factors” OR “Factors affecting” AND Ethiopia		604	March 25, 2023	Google Scholar
				40	March 31, 2023	Google

Table Supl. 2: Quality assessment for the included studies using Newcastle Ottawa quality assessment form for cohort studies

Newcastle Ottawa quality assessment and its weight		Included studies and their scores for each checklist																	
**Selection**	**Star (*)**	Gemechu et al, 2022	Atalell et al. 2018	Chanie et al. 2021	Nigusie et al 2021	Dawit, 2021	Abrha H, et al. 2015	Birhan et al 2021	Gezae, et al., 2020	Habtegiorgis 2018	Kassa et al 2012	Lelisho 2022	Lelisho 2022	Hailu 2013	Seyoum et al 2022	Sime et al 2022	Teklu 2017	Wondimu 2019	Sileshi etal, 2013
1)	Representativeness of the exposed cohort each one star																		
Truly representative	1	*	*	*	*	*		*	*		*	*	*	*	*	*	*	*	*
Somewhat representative	1						*			*									
Selected group	1																		
No description	1																		
2)	**Selection of the non-exposed cohort each one star**																		
Drawn from the same community as the exposed cohort	1	*	*	*	*	*	*	*	*	*	*	*	*	*	*	*	*	*	*
Drawn from a different source	1																		
No description of the derivation of the non-exposed cohort	1																		
3	**Ascertainment of exposure each one star**																		
Secure record	1	*	*	*	*	*	*	*	*	*	*	*	*	*	*	*	*	*	*
Structured interview	1																		
Written self-report																			
No description																			
Other																			
4)	**Demonstration that outcome of interest was not present at start of study**																		
Yes	1	*	*	*	*		*	*	*	*	*	*	*	*	*	*	*	*	*
No																			

**Comparability**																			
1) **Comparability of cohorts on the basis of the design or analysis controlled for confounders**																			
The study controls for age, sex and marital status	1	0	*	*	0	*	*	*	0	*	*	*	*	0	*	*	*	*	*
Study controls for other factors O	1	*	*	*	*	*	*	*	*	*	*	*	*	*	*	*	*	*	*
Cohorts are not comparable on the basis of the design or analysis controlled for confounders																			
**Outcome**																			
1)	**Assessment of outcome**																		
Independent blind assessment	1																		
Record linkage	1	*	*	*	*	*	*	*	*	*	*	*	*	*	*	*	*	*	*
Self-report																			
No description																			
Other																			
2) **Was follow-up long enough for outcomes to occur**																			
Yes	1	*	*	*	*	*		*	*	*	*	*	*	*	*	*	*	*	*
No							√												
Indicate the median duration of follow-up		4.5 y	4.0 y	6.86 y	5 y	6 y	0.93 y	2.5 ye	3.08 year	1.88 y	2.55	2.5 y	4.0 y	2.28 y	4.0 y	2.26y	2.96 y	5.0 y	0.55 Y
3) **Adequacy of follow-up of cohorts**																			
Complete follow-up all subject accounted																			
Subjects LTFU unlikely to introduce bias- number lost		*	*	*	*	*					*	*	*		*	*			*

less than or equal to 20% or description of those lost suggested no different from those followed.																			
Follow up rate less than 80% and no description of those lost																			
No statement							√	√	√	√				√			√	√	
**Additional parameters**																			
**1. Sample size (Maximum 1)**																			
Justified and satisfactory	1	*	*		*	*	*	*	*	*	*	*	*	*	*	*	*	*	*
Adequately powered to detect a difference		*	*	*	*	*	*	*	*	*	*	*	*	*	*	*	*	*	*
Not justified																			
**2. Statistical test (Maximum 1)**																			
clearly described and appropriate		*	*	*	*	*	*	*	*	*	*	*	*	*	*	*	*	*	*
is not appropriate, not described or incomplete																			
**3. Clear variables (Maximum 2)**																			
Both variables clearly defined including time period		*	*	*	*	*	*		*	*	*	*	*	*	*	*	*	*	*
One or another variable clearly defined								*											

Neither variable clearly defined																			
**Quality of studies**		Good	Good	Good	Good	Goo<br>d	Good	Good	Good	Good	Good	Good	Good	Good	Good	Good	Goo<br>d	Good	Good

❖- *Clinical factors*

Thresholds for converting the Newcastle-Ottawa scales to AHRQ standards (good, fair, and poor):

1. **Good quality:** 3 or 4 stars in selection domain AND 1 or 2 stars in comparability domain AND 2 or 3 stars in outcome/exposure domain
2. **Fair quality:** 2 stars in selection domain AND 1 or 2 stars in comparability domain AND 2 or 3 stars in outcome/exposure domain
3. **Poor quality:** 0 or 1 star in selection domain OR 0 stars in comparability domain OR 0 or 1 stars in outcome/exposure domain

Table Supl.3: PRISMA 2020 checklist

Section and Topic	Item #	Checklist item	Location where item is reported
**TITLE**			
Title	1	Identify the report as a systematic review.	Page -1, lines 1–3
**ABSTRACT**			
Abstract	2	See the PRISMA 2020 for Abstracts checklist.	Page-2, lines 25–49
**INTRODUCTION**			
Rationale	3	Describe the rationale for the review in the context of existing knowledge.	Page 4, lines 87–95
Objectives	4	Provide an explicit statement of the objective(s) or question(s) the review addresses.	Page-4, lines 96–97
**METHODS**			
Eligibility criteria	5	Specify the inclusion and exclusion criteria for the review and how studies were grouped for the syntheses.	Page- 4 and 5, lines 107–113
Information sources	6	Specify all databases, registers, websites, organisations, reference lists and other sources searched or consulted to identify studies. Specify the date when each source was last searched or consulted.	Page-4, lines 101–103

Section and Topic	Item #	Checklist item	Location where item is reported
Search strategy	7	Present the full search strategies for all databases, registers and websites, including any filters and limits used.	Page-6, lines 145–157
Selection process	8	Specify the methods used to decide whether a study met the inclusion criteria of the review, including how many reviewers screened each record and each report retrieved, whether they worked independently, and if applicable, details of automation tools used in the process.	Page 5, lines 119–122
Data collection process	9	Specify the methods used to collect data from reports, including how many reviewers collected data from each report, whether they worked independently, any processes for obtaining or confirming data from study investigators, and if applicable, details of automation tools used in the process.	Page 5, lines 122–126
Data items	10a	List and define all outcomes for which data were sought. Specify whether all results that were compatible with each outcome domain in each study were sought (e.g., for all measures, time points, analyses), and if not, the methods used to decide which results to collect.	Page 5, lines 115–116
	10b	List and define all other variables for which data were sought (e.g., participant and intervention characteristics, funding sources). Describe any assumptions made about any missing or unclear information.	Page-5-line 117
Study risk of bias assessment	11	Specify the methods used to assess risk of bias in the included studies, including details of the tool(s) used, how many reviewers assessed each study and whether they worked independently, and if applicable, details of automation tools used in the process.	Page-5 lines 123–126<br>Page 6, line 143
Effect measures	12	Specify for each outcome the effect measure(s) (e.g., risk ratio, mean difference) used in the synthesis or presentation of results.	Page 5, lines 115–118
Synthesis methods	13a	Describe the processes used to decide which studies were eligible for each synthesis (e.g., tabulating the study intervention characteristics and comparing against the planned groups for each synthesis (item #5)).	Page 5, lines 120–123
	13b	Describe any methods required to prepare the data for presentation or synthesis, such as handling of missing summary statistics, or data conversions.	Page 5, lines 123–127
	13c	Describe any methods used to tabulate or visually display results of individual studies and syntheses.	Page 5, lines 139–141
	13d	Describe any methods used to synthesize results and provide a rationale for the choice(s). If meta-analysis was performed, describe the model(s), method(s) to identify the presence and extent of statistical heterogeneity, and software package(s) used.	Page 5, lines 132–139
	13e	Describe any methods used to explore possible causes of heterogeneity among study results (e.g., subgroup analysis, meta-regression).	Page 6, lines 141–142
	13f	Describe any sensitivity analyses conducted to assess robustness of the synthesized results.	Page 6, line 143
Reporting bias assessment	14	Describe any methods used to assess risk of bias due to missing results in a synthesis (arising from reporting biases).	Page 6, lines 144–145

Section and Topic	Item #	Checklist item	Location where item is reported
Certainty assessment	15	Describe any methods used to assess certainty (or confidence) in the body of evidence for an outcome.	Page-5, line 140
**RESULTS**			
Study selection	16a	Describe the results of the search and selection process, from the number of records identified in the search to the number of studies included in the review, ideally using a flow diagram.	Page 6, lines 148–161 and Fig 1-PRISMA
	16b	Cite studies that might appear to meet the inclusion criteria, but which were excluded, and explain why they were excluded.	Page 6, lines 156–157
Study characteristics	17	Cite each included study and present its characteristics.	Page 6, Line 158
Risk of bias in studies	18	Present assessments of risk of bias for each included study.	Page 6, lines 163–169, table supl.2
Results of individual studies	19	For all outcomes, present, for each study: (a) summary statistics for each group (where appropriate) and (b) an effect estimates and its precision (e.g., confidence/credible interval), ideally using structured tables or plots.	a) Page 7, lines 171–181; Table 1<br>b) Page 7, lines 185–192; Table 2, Figure 2
Results of syntheses	20a	For each synthesis, briefly summarise the characteristics and risk of bias among contributing studies.	Table 1, and Table Supl. 2 (supplementary file)
	20b	Present results of all statistical syntheses conducted. If meta-analysis was done, present for each the summary estimate and its precision (e.g. confidence/credible interval) and measures of statistical heterogeneity. If comparing groups, describe the direction of the effect.	Page 7, lines 187–190; Table 2 and Figure 2
	20c	Present results of all investigations of possible causes of heterogeneity among study 199results.	Pages 7–8, lines 196–216; Tables Supl.4 and 5; Figure Supl. 1 and 2
	20d	Present results of all sensitivity analyses conducted to assess the robustness of the synthesized results.	Page 8, 223–226; Table Supl. 6 and Figure Supl. 3
Reporting biases	21	Present assessments of risk of bias due to missing results (arising from reporting biases) for each synthesis assessed.	Page 9, lines 228–230; Table Supl. 5
Certainty of evidence	22	Present assessments of certainty (or confidence) in the body of evidence for each outcome assessed.	Tables 2 and 5
**DISCUSSION**			
Discussion	23a	Provide a general interpretation of the results in the context of other evidence.	Pages 10–12, lines 259–328
	23	Discuss any limitations of the evidence included in the review.	Pag12, lines 333–355

Section and Topic	Item #	Checklist item	Location where item is reported
	b		
	23c	Discuss any limitations of the review processes used.	Page 12, lines 333–355
	23d	Discuss implications of the results for practice, policy, and future research.	Page 13, lines 343–346
**OTHER INFORMATION**			
Registration and protocol	24a	Provide registration information for the review, including register name and registration number, or state that the review was not registered.	It was not registered
	24b	Indicate where the review protocol can be accessed, or state that a protocol was not prepared.	It was not prepared
	24c	Describe and explain any amendments to information provided at registration or in the protocol.	It was not registered
Support	25	Describe sources of financial or non-financial support for the review, and the role of the funders or sponsors in the review.	Page 13, line 363
Competing interests	26	Declare any competing interests of review authors.	Page 13, line 365
Availability of data, code and other materials	27	Report which of the following are publicly available and where they can be found: template data collection forms; data extracted from included studies; data used for all analyses; analytic code; any other materials used in the review.	Page 13 lines 367–369

***Source**: Page MJ, McKenzie JE, Bossuyt PM, Boutron I, Hoffmann TC, Mulrow CD, et al. The PRISMA 2020 statement: an updated guideline for reporting systematic reviews. BMJ 2021;372: n71. Doi: 10.1136/bmj. n71*

Table Supl.4: Subgroup-analysis of mortality rate among TB/HIV coinfected patients in Ethiopia by place of region from 2010 to 2023.

Study region	Author (year)	Effect	95% CI	Wgt%	Q-Stat	I^2 %	P-value
SNNPR	Gemechu et al (2022)	2.78	0.78–4.79	6.04			
	Dawit (2021)	2.97	0.84–5.11	6.02			
	Lelisho ME (2022)	10.47	5.86–15.07	5.50			
	Lelisho ME (2022)	14.77	9.49–20.05	5.31			
	Wondimu (2019)	61.06	53.00–69.12	4.48			
	Subgroup, DL	17.64	6.78–28.50	27.35	211.05	98.1	<0.001
Amhara	Atalell et al. (2018)	3.25	0.94–5.57	5.99			
	Chanie et al. (2021)	3.67	1.12–6.22	5.95			
	Birhan et al (2021)	16.69	11.17–22.20	5.25			
	Sileshi, B. (2013)	49.75	42.09–57.41	4.60			
	Subgroup, DL	17.72	5.12–30.32	21.79	147.95	98	<0.001
Tigray	Nigusie J. et al (2021)	18.01	12.35–23.68	5.20			
	KMGezae, et al., (2020)	7.14	3.29–10.99	5.69			
	Subgroup, DL	12.37	1.72–23.02	10.89	9.68	89.7	<0.01
Oromia	Abrha H, et al. (2015)	21.71	15.68–27.74	5.09			
	Hailu (2013)	9.59	5.16–14.02	5.54			
	Subgroup, DL	15.47	3.60–27.34	10.64	10.06	90.1	<0.01
Dire-Dawa	Habtegiorgis (2018)	8.36	4.20–12.52	5.61			
	Subgroup, DL	8.36	4.20–12.52	5.61	0	-	-
Addis Ababa	A. Kassa et al (2012)	2.80	0.78–4.82	6.04			
	Seyoum et al (2022)	1.22	0.83–1.60	6.17			
	Subgroup, DL	1.69	0.27–3.10	12.21	2.29	56.5	0.13
Harar & Dire-	Sime et al (2022)	11.31	6.55–16.06	5.46			
Dawa	Subgroup, DL	11.31	6.55–16.06	5.46	0	-	-
7 hospitals in 5 regions	Teklu (2018)	2.60	0.73–4.47	6.06			
	Subgroup, DL	2.60	0.73–4.47	6.06	0	-	-
National	Overall death rate/100pyo	12.49	9.24–15.74	100.00	552.18	96.9	<0.001

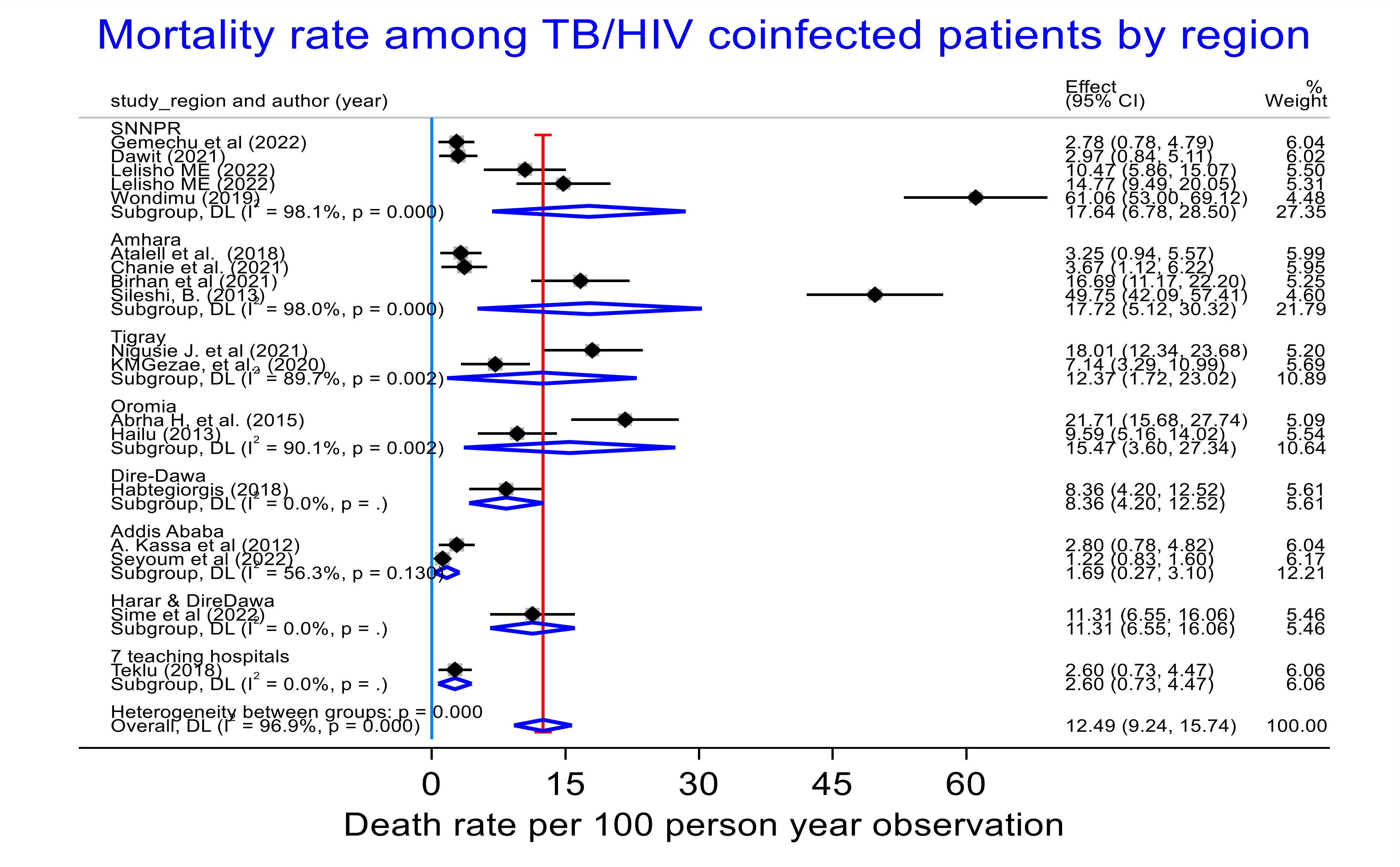

Figure Sup1. 1: Sub-group analysis of the mortality rate by region among TB/HIV coinfected patients in Ethiopia

Table Supl. 5: Sub-group analysis of mortality rate among TB/HIV coinfected patients by sample size category in the primary studies

Sample Size Cat	Author (Year)	Effect	95% CI	Weight %	Cochran's Q Stat	I²%	P-value
less than 500	Gemechu et al (2022)	2.78	0.78–4.79	6.04			
	Atalell et al. (2018)	3.25	0.94–5.57	5.99			
	Chanie et al. (2021)	3.67	1.12–6.22	5.95			
	Nigusie J. et al (2021)	18.01	12.35–3.68	5.20			
	Dawit (2021)	2.97	0.84–5.11	6.02			
	Abrha H, et al. (2015)	21.71	15.68–27.74	5.09			
	Birhan et al (2021)	16.69	11.17–22.20	5.25			
	Gezae, et al., (2020)	7.14	3.29–10.99	5.69			
	Habtegiorgis (2018)	8.36	4.20–12.52	5.61			
	A. Kassa et al (2012)	2.80	0.78–4.82	6.04			
	Lelisho ME (2022)	10.47	5.86–15.07	5.50			
	Lelisho ME (2022)	14.77	9.49–20.05	5.31			
	Wondimu (2019)	61.06	53.00–69.12	4.48			
	Sileshi, B. (2013)	49.75	42.09–57.41	4.60			
	Subgroup, DL	15.11	10.08–20.13	76.77	413.71	96.9	<0.001
500 and more	Hailu (2013)	9.59	5.16–14.02	5.54			
	Seyoum et al (2022)	1.22	0.83–1.60	6.17			
	Sime et al (2022)	11.31	6.55–16.06	5.46			
	Teklu (2018)	2.60	0.73–4.47	6.06			
	Subgroup, DL	5.36	1.83–8.88	23.23	32.29	90.7	<0.001
Overall incidence of mortality		12.49	9.24–15.74	100.00	552.18	96.9	<0.001

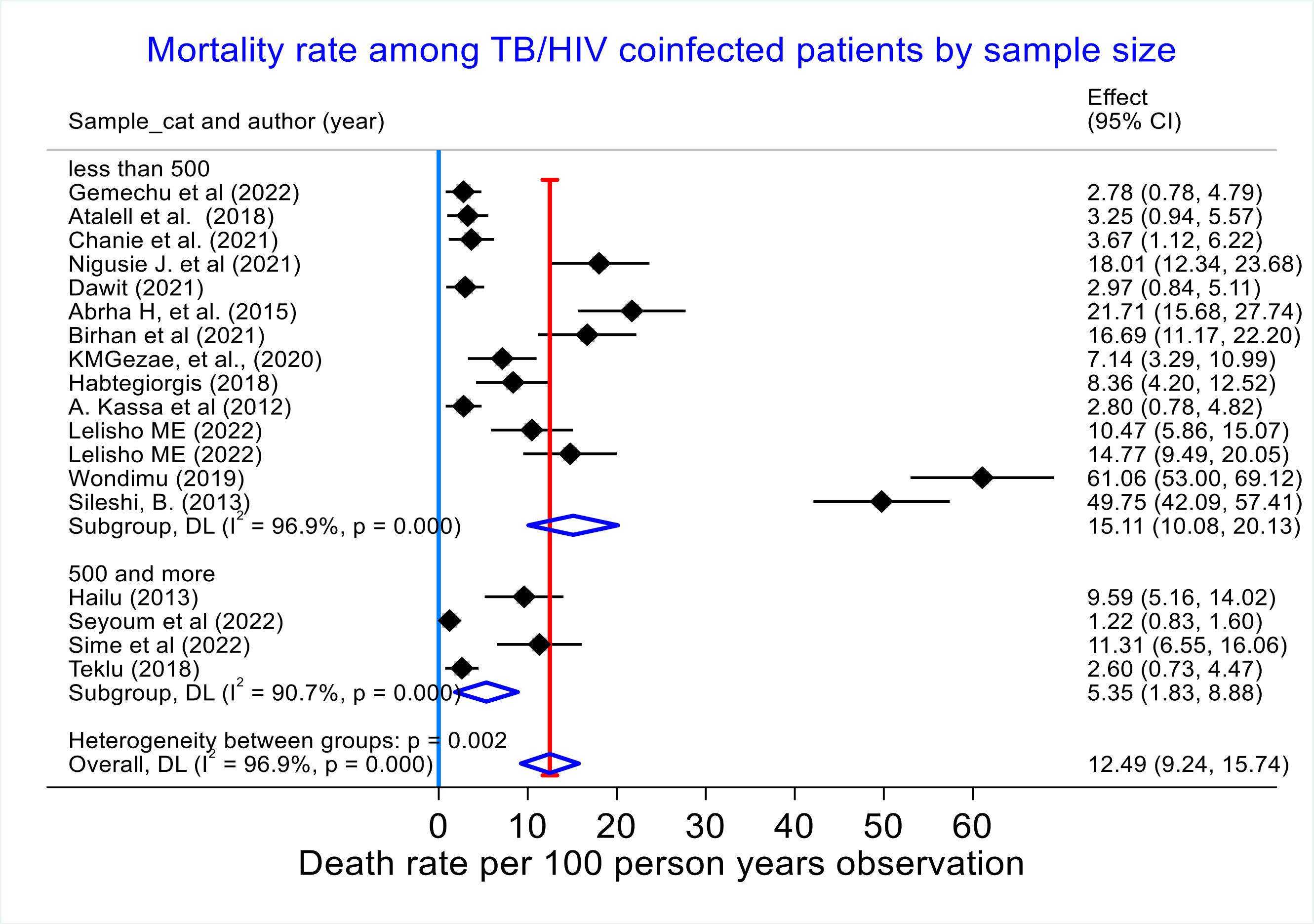

Figure Supl. 2. Sub-group analysis of the mortality rate by sample size category among TB/HIV coinfected patients in Ethiopia

Table Supl. 6: Estimated pooled mortality rate among TB/HIV-coinfected patients in Ethiopia after sensitivity analysis

Author (year)	Effect	95% CI	Wt.%	C/ Q stat	I² %	P-value
Gemechu et al (2022)	2.78	0.78–4.79	6.25			
Atalell et al. (2018)	3.25	0.94–5.57	6.22			
Chanie et al. (2021)	3.67	1.12–6.22	6.20			
Nigusie J. et al (2021)	18.01	12.35–23.68	5.66			
Dawit (2021)	2.97	0.84–5.11	6.24			
Abrha H, et al. (2015)	21.71	15.68–27.74	5.58			
Birhan et al (2021)	16.69	11.17–22.202	5.69			

KMGezae, et al., (2020)	7.14	3.29–10.99	6.01			
Habtegiorgis (2018)	8.36	4.20–12.52	5.96			
A. Kassa et al (2012)	2.80	0.78–4.82	6.25			
Lelisho ME (2022)	10.47	5.86–15.07	5.88			
Lelisho ME (2022)	14.77	9.49–20.05	5.74			
Hailu (2013)	9.59	5.16–14.02	5.91			
Sime et al (2022)	11.31	6.55–16.06	5.85			
Wondimu (2019)	61.06	53.00–69.12	5.10			
Teklu (2018)	2.60	0.73–4.47	6.26			
Sileshi, B. (2013)	49.75	42.09–57.41	5.20			
Overall, DL	13.62	9.53–17.72	100.00	433.99	96.3,95% CI: 88–98.2	<0.001

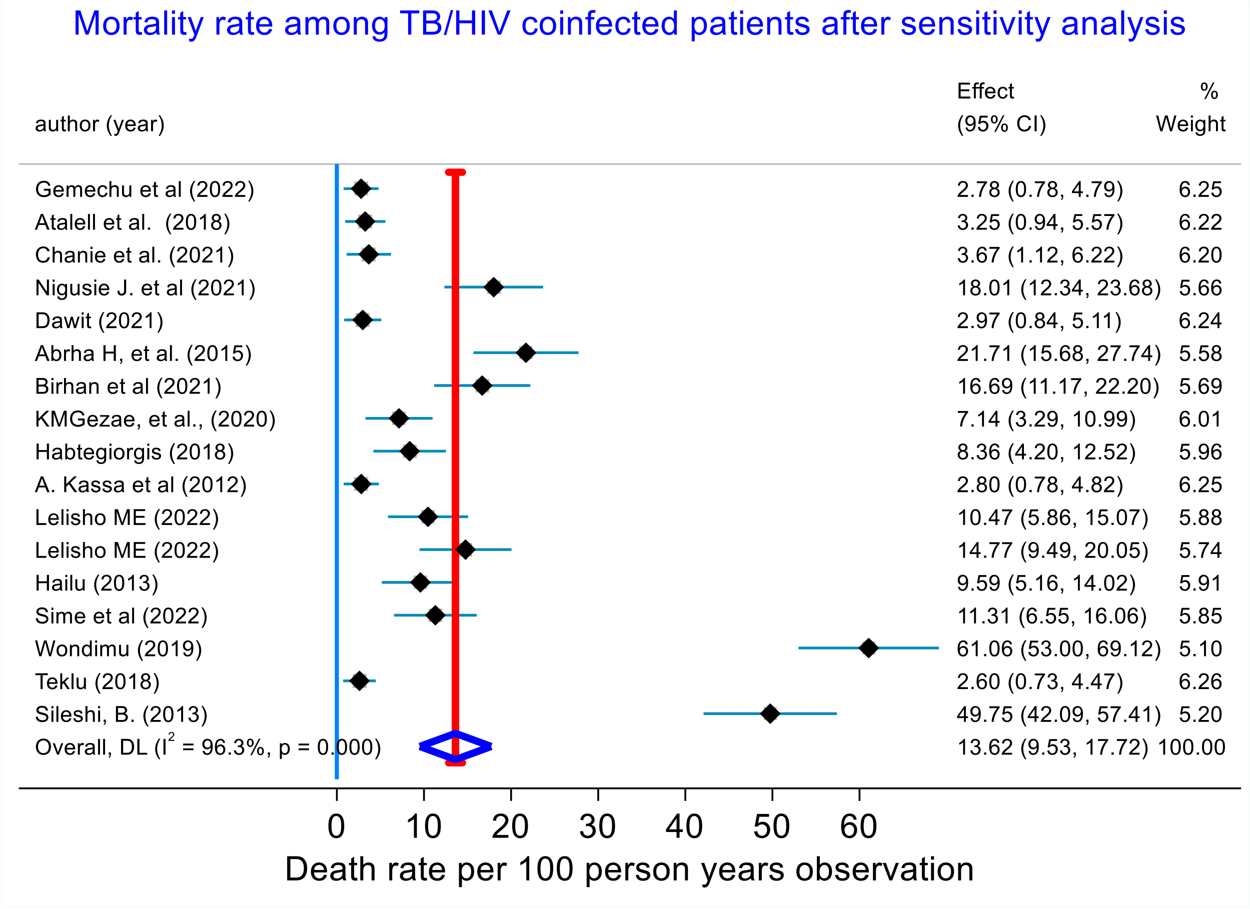

Figure Supl. 3: Forest plot showing mortality rate among TB/HIV coinfected patients in Ethiopia after leave-one-out sensitivity analysis

Table Supl. 7: Small study effect or publication bias

Study Effect	Coefficient	Standard error	t-test	P-value	95% CI
Slope	-0.33	0.76	-0.44	0.669	-1.95–1.29
Bias	5.60	1.04	5.40	<0.001	3.4–7.80
